# Correlation between the Porosity and Permeability of a Polymer Filter Fabricated via CO_2_-Assisted Polymer Compression

**DOI:** 10.3390/membranes10120391

**Published:** 2020-12-03

**Authors:** Takafumi Aizawa, Yoshito Wakui

**Affiliations:** Research Institute for Chemical Process Technology, National Institute of Advanced Industrial Science and Technology, 4-2-1 Nigatake, Miyagino-ku, Sendai 983-8551, Japan; y-wakui@aist.go.jp

**Keywords:** CO_2_-assisted polymer compression, filter, gas permeability, porosity, pore diameter

## Abstract

A porous filter was fabricated by plasticizing polymer fibers with CO_2_, followed by pressing and adhering; then, its gas permeability, a basic physical property of filters, was measured using N_2_. The as-obtained filter was well compressed and expected to approximate a sintered porous material. Therefore, the fabricated filter was analyzed by applying the Darcy law, and the correlation between its gas permeability and porosity was clarified. The gas permeability decreased owing to both pore size and porosity reduction upon increasing the degree of compression, which is a feature of the CO_2_-assisted polymer compression method. In particular, without any contradiction of pore size data previously reported, the gas permeability was clearly determined by the filter porosity and pore size. This study can serve as a guide for designing filters via CO_2_-assisted polymer compression.

## 1. Introduction

Polymers are indispensable in modern society and are used everywhere [1,2]; they can be easily converted into fibers and applied in various ways, including in clothes, filters, and wipers, with the advantages of their inexpensiveness, lightness, and strength [3,4]. In particular, their use as filters, based on the gap between polymer fibers, has been recently attracting attention and is actively studied due to the air pollution problem related to <2.5 µm diameter particular matter (PM2.5) [5,6]. Porous membranes made of polymer fibers have been tested as face masks [7], air filters [8], battery separators [9], and so on.

Polymers are plasticized via CO_2_ impregnation [10,11]; in particular, the amount of supercritical CO_2_ impregnated into polymers has been reported [12,13,14]. We developed a CO_2_-assisted polymer compression (CAPC) method, which consists of the CO_2_ plasticization of fibrous sheets that are successively pressed to obtain porous polymer materials [15]. It uses only nontoxic CO_2_, which is released into the atmosphere after molding and, thus, does not remain in the material; therefore, the as-obtained products are suitable also for application fields requiring no contamination, such as the medical and food industries. The bonding mechanism of this method is point bonding at the portion where the fibers overlap. A comparison between the measured adhesive strength and a model based on its average value per point and the number of adhesive points has demonstrated consistency between simulated and experimental results [16]. Moreover, in this method, the porosity and pore diameter can be controlled by the amount of raw fiber material and the pressing position [17]; via this porosity control, a drug-loaded tablet has been fabricated, successfully changing sustained drug release [18]. As for mass production, the productivity of this method can be improved by simultaneously preparing several samples with a single press [19]. A multi-layered porous structure with varying porosity has been fabricated using CAPC treatment in two steps [20].

The basic properties such as gas permeability, degree of particle trapping, and lifetime are crucial for filter applications. However, because the CAPC method is a relatively new technique, their roles and potential values in this process are unknown. Thus, to elucidate the basic characteristics of a porous filter fabricated via CAPC, this study focused on gas permeability, especially by experimentally measuring the N_2_ permeability, and on its relationship with porosity.

## 2. Materials and Methods

**Figure 1:**
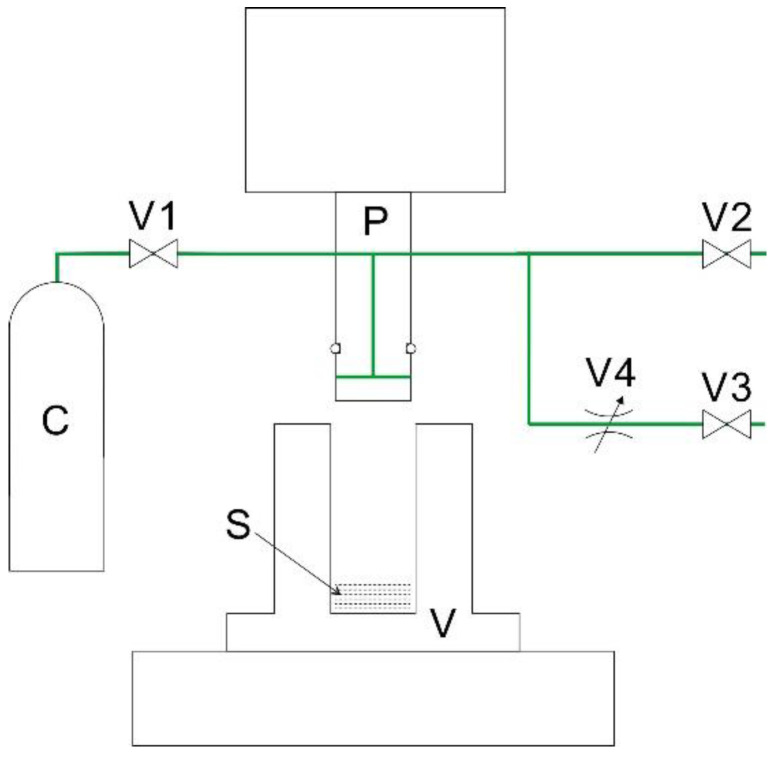
Schematic of the CO_2_-assisted polymer compression equipment. C, CO_2_ cylinder; P, piston; S, sample; V, high-pressure vessel; V1, introduction valve; V2, exhaustion valve for rapid exhaustion; V3, exhaustion valve for slow exhaustion; V4, metering valve.

Figure 1 demonstrates the CAPC equipment used in this study. From the findings of the numbering-up experiment [19], the two-step exhaust process was suggested as an important factor to ensure the uniformity of the sample; therefore, we updated the exhaust line reported in previous studies [15,16,17,18]. A nonwoven fabric (fiber diameter: 4 or 8 μm; basis weight: 30 g m^−2^) was supplied by Nippon Nozzle Co., Ltd. (Kobe, Japan) by using polyethylene terephthalate pellets (TK3, Bell Polyester Products Inc., Yamaguchi, Japan). The nonwoven fabric was punched into a diameter of 30 mm by a punch, and different numbers of sheets (10 for 0.219 g, 12 for 0.263 g, 14 for 0.306 g, 16 for 0.350 g, 18 for 0.394 g, and 20 for 0.438 g) were prepared by adjusting the weight.

The CAPC process was conducted as follows. The experiments were performed at room temperature. A predetermined number of raw material sheets were placed in a high-pressure vessel with an inner diameter of 32.0 mm, below a piston with an outer diameter of 31.5 mm. Then, the piston was lowered into the vessel, which was sealed by an O-ring placed around the piston itself. After closing the exhaustion valve, V2, the introduction valve, V1, was opened to inject gaseous CO_2_ from the CO_2_ cylinder; next, V1 was closed and V2 was opened, removing CO_2_ together with the air remaining from the beginning. By repeating this operation three times, the air in the high-pressure vessel was almost replaced by CO_2_. Thereafter, by closing V2 and then opening V1, additional gaseous CO_2_ was introduced at vaper pressure (6 MPa) in the vessel and trapped by successively closing V1. Then, the sample was compressed to a specified thickness by lowering the piston to the press position; after 10 s of compression, the exhaustion valve, V3, was opened for 30 s to slowly release CO_2_ through the metering valve (SS-SS1, Swagelok Inc., Solon, Ohio, United States of America) and V2 was opened to instantly let CO_2_ out into the atmosphere. Finally, the piston we lifted and the CAPC product were taken out to complete the CAPC process. In this method, the polymer is plasticized by CO_2_ impregnation, and then its shape is fixed via compression and CO_2_ removal while kept in a compressed state. Therefore, the press position is directly reflected in the product thickness. In this study, the press position was adjusted so as to obtain a product thickness of 0.6 mm, which was measured with a micrometer caliper. Since the thickness of the sample was uniform, a larger number of nonwoven fabric sheets resulted in a higher density and a lower porosity of the resulting product.

The porosity was calculated from the weight of the sample and the volume and density of the polymer used. The thickness, *L*_solid_, of a cylinder with an outer diameter of 30.0 mm and no voids can be calculated based on the outer diameter of the constituent nonwoven fabric, sample weight, and polyethylene terephthalate density (1.34 g mL^−1^) given in the datasheet provided by the supplier, which was 0.231, 0.278, 0.323, 0.370, 0.416, and 0.463 mm for 0.219, 0.263, 0.306, 0.350, 0.394, and 0.438 g, respectively. In our experiment, *L*_porous_, which was measured with a micrometer caliper, was higher than *L*_solid_ because of the pores. Then, its porosity, *α*, can be derived from the calculated *L*_solid_ and its real thickness *L*_porous_ as follows:

(1) $$\alpha =\frac{L_{\mathrm{porous}}-L_{\mathrm{solid}}}{L_{\mathrm{porous}}}$$

To obtain an outer diameter of 25 mm, which is required to measure the gas permeability, the CAPC product was punched out to 25 mm.

**Figure 2:**
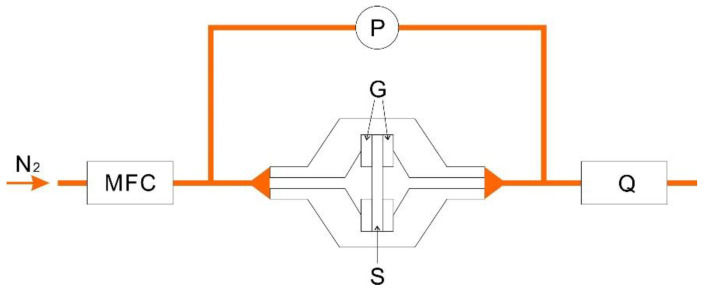
Schematic of the gas permeability measurements. G, gaskets; MFC, mass flow controller; P, differential pressure gauge; Q, flowmeter; S, sample.

Figure 2 shows the configuration of the gas permeability measurements. Each sample, sandwiched between gaskets (inner diameter: 8.11 mm), was placed in a holder of 25 mm. The flow rate of N_2_ gas was controlled by a mass flow controller (SEC-E40 and PAC-D2, HORIBA, Ltd., Kyoto, Japan). The increment in the accuracy flow rate was measured by a soap film flowmeter (SF-1 and VP-2, HORIBA, Ltd., Kyoto, Japan). The pressure loss was evaluated with a differential pressure gage. A DPG-01U differential pressure gage (Custom Co., Chiyoda-ku, Japan) was utilized for all of the samples except for the one obtained by laminating 20 sheets with a fiber diameter of 4 μm. In the case of laminating 20 sheets with a fiber diameter of 4 μm, the large pressure loss of the sample exceeded the measurement range of the DPG-01U instrument and, thus, an HT-1500NH differential pressure gage (Hodaka Co., Ltd., Osaka, Japan) was used. Nine samples of each product type were analyzed under the same conditions.

The surface of the samples was observed with a TM-1000 scanning electron microscopy (SEM) system (Hitachi High-Technologies Co., Minato-ku, Japan).

## 3. Results and Discussion

**Figure 3:**
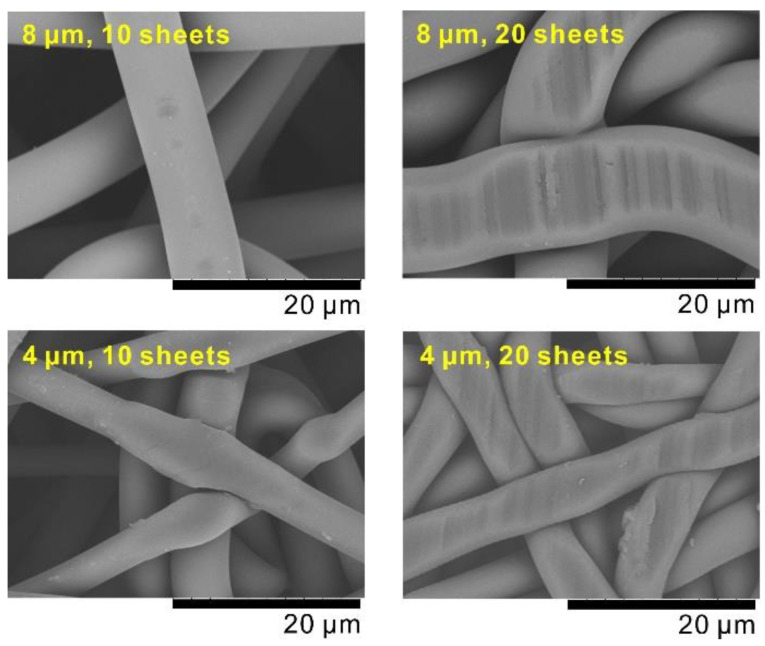
Scanning electron microscopy images of the surface of the samples fabricated via CO_2_-assisted polymer compression.

Figure 3 shows the SEM images of the surface of the as-prepared samples, revealing traces of crushing on the fiber surface, probably owing to the bottom stainless steel surface of the piston. The part where the fibers were not crushed into one another did not look different to the raw nonwoven fabric, and no swelling or foaming was observed. Because the sample thickness was uniform, compared to the product with 10 laminated sheets (with fibers of 8 μm in diameter), the product with 20 laminated sheets of the same fibers exhibited larger crushing and thickening traces; although the fibers were crushed, there were spaces between them, which became pores. The pore size of the product with 20 laminated sheets was smaller than that with 10 laminated sheets. As for the fiber sheets of 4 µm in diameter, the fiber surface was more evidently crushed than that of the fiber sheets of 8 µm in diameter. Regarding the pores, the SEM images suggest that a porous body consisting of small-diameter fibers also has small-diameter pores. As when using fiber sheets of a diameter of 8 μm in diameter, the pore size of the product with more laminated samples was smaller than that with less laminated samples.

**Figure 4:**
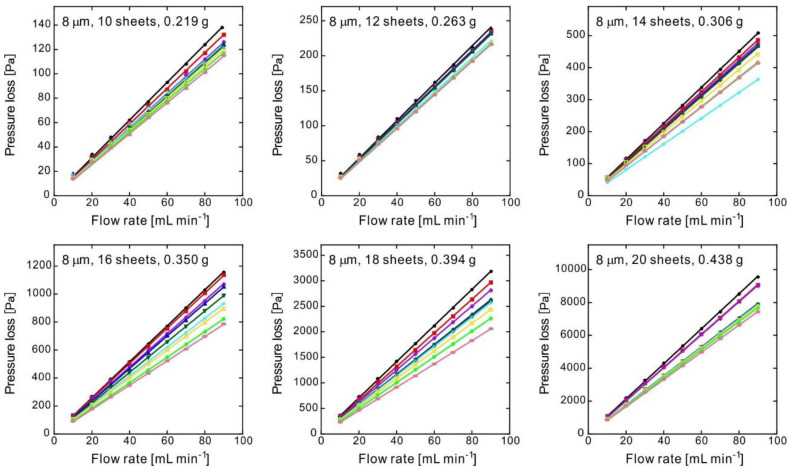
Pressure loss as a function of the flow rate for various 0.6-mm-thick samples consisting of nonwoven fabric with a fiber diameter of 8 μm and a different number of sheets, obtained via CO_2_-assisted polymer compression. The straight lines passing through the origin of the graphs represent the fitting of the experimental results; each graph shows the results for nine samples of the corresponding product type.

Figure 4 illustrates the pressure loss for the products obtained from the raw fibers of 8 μm in diameter; each experimental value was in line with the corresponding fitting curve. Because the linearity of the pressure loss of each sample was preserved, it is considered that the N_2_ permeability was accurately measured. The variety of the slope might have been caused by sample non-uniformity. The nonwoven fabric used as the raw material had a basis weight of 30 g m^−2^, but there was local non-uniformity of the nonwoven fabric fabricated by the melt blown method.

A porous material formed by pressing a fibrous material should have a structure similar to that of a sintered filter in terms of pore connection. Therefore, the samples were analyzed similarly to sintered filters. The following relationship, known as the Forchheimer equation, is normally established between the pressure loss (Δ*P*) in the filter, the filter thickness (*L*), and the flow velocity (*v*_s_) [21,22]:

(2) $$\frac{\Delta P}{L}=\frac{\mu v_{s}}{k_{1}}+\frac{\rho v_{s}^{2}}{k_{2}}$$

where *μ* is the viscosity, *ρ* is the density, and *k*_1_ and *k*_2_ are the Darcian and non-Darcian permeability coefficients, respectively. The first term represents the laminar flow effect and the second indicates the turbulence effect. In the case of a compressible fluid such as a gas, based on the compression in the porous body, Equation (2) can be written as:

(3) $$\frac{P_{i}^{2}-P_{o}^{2}}{2P_{o}L}=\frac{\mu v_{s}}{k_{1}}+\frac{\rho v_{s}^{2}}{k_{2}}$$

where *P_i_* and *P_o_* are the inlet and outlet pressures, respectively [23,24]. When the pressure loss is low (as in this study), it approximately matches Equation (2). Flows with the same cross-sectional area exhibit a proportional relationship between flow rate and velocity. The well-fitting linear approximation shown in Figure 4 indicates that the turbulence effect was negligible. In fact, we attempted fitting by Equation (2), but a significant coefficient of the second term could not be obtained; this means that the flow rate was low and the flow inside the porous material could only be laminar.

**Figure 5:**
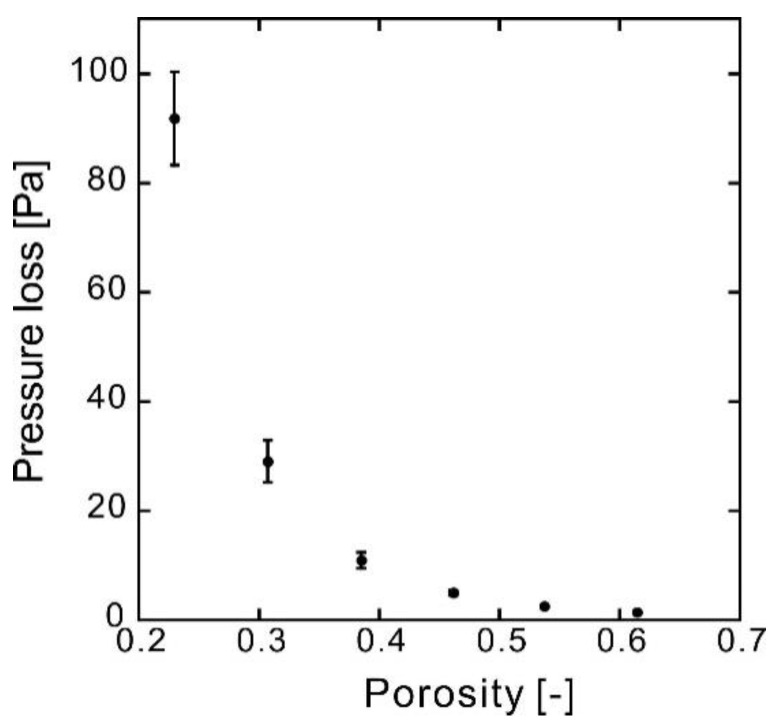
Pressure loss at 1 mL min^−1^ as a function of porosity for the samples made of nonwoven fabric with a fiber diameter of 8 μm, obtained via CO_2_-assisted polymer compression. Error bars indicate standard deviations.

Figure 4 plots the flow rates and pressure losses of the samples; the slopes represent the pressure drop when flowing at 1 mL min^−1^. Figure 5 shows the average slopes of the experiments shown in Figure 4, along with the average porosity experimentally measured.

The experimental data are summarized in Table 1. According to Figure 5 and Table 1, for the same flow rate and sample thickness, the pressure loss decreased rapidly as the porosity increased; these experimental results are reasonable.

Since the inner diameter of both the gaskets holding the samples was 8.11 mm, the gas flowed through a 0.516 cm^2^ cross-sectional area of the porous body at a velocity of 3.23 × 10^−4^ m s^−1^ when the flow rate was 1 mL min^−1^. Given the low pressure drop of the soap film flowmeter (42 Pa at 90 mL min^−1^), the outlet pressure of the filter was almost equal to the atmospheric pressure. By considering an N_2_ viscosity of 1.85 × 10^−5^ Pa s at room temperature and under atmospheric pressure [25], we derived the corresponding *k*_1_ values (Table 1).

The *k*_1_ parameter includes the porosity effect, the pore diameter, and the flow path tortuosity. For a sintered filter, the pore diameter can often be estimated via an Ergun-like relationship as follows [26,27]:

(4) $$d_{\mathrm{pore}}=\sqrt{\frac{150k_{1}}{2.25\alpha }}$$

We derived it from the *k*_1_ values shown in Table 1, finding pore diameters of 17 and 3.4 μm for a porosity of 62% and 23%, respectively.

In a previous study on the relationship between porosity and pore size, pore size was shown to decrease with decreasing porosity [17], which is consistent with the results shown in Table 1. This relationship is clear from the SEM image in Figure 3. A mercury porosimetry analysis revealed pore sizes of 5 and 6 µm at a porosity of 25% and 32%, respectively [17]. The values in Table 1 do not differ significantly from these results and the analysis seems reasonable; this means that the gas permeability of the CAPC-fabricated products can be explained by their porosity and pore diameter.

**Figure 6:**
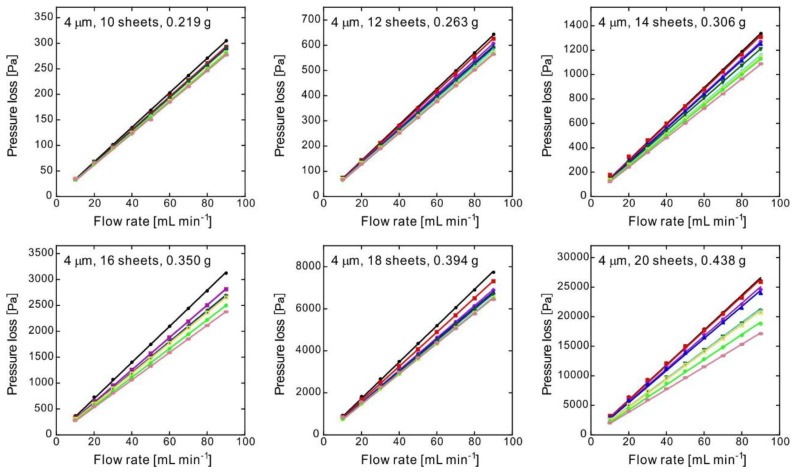
Pressure loss as a function of the flow rate for various 0.6-mm-thick sample consisting of nonwoven fabric with a fiber diameter of 4 μm and a different number of sheets, obtained via CO_2_-assisted polymer compression. The straight lines passing through the origin of the graphs represent the fitting of the experimental results; each graph shows the results for nine samples of the corresponding product type.

Figure 6 and Table 2 show the results for a nonwoven fabric with fibers of 4 μm in diameter. In particular, Figure 6 illustrates the data for the samples with the same porosity as those in Figure 4, indicating a larger pressure loss compared to that in the samples with fibers of 8 μm in diameter. A comparison between Table 1 and Table 2 clearly reveals this tendency. In this case, the pressure loss was approximately 2.3–2.8 times larger. Table 2 summarizes the pore diameters calculated as for Table 1, showing values approximately 0.60–0.66 times those of the samples with fibers of 8 μm in diameter. This is consistent with the state of the pores observed by the SEM analysis (Figure 3).

**Figure 7:**
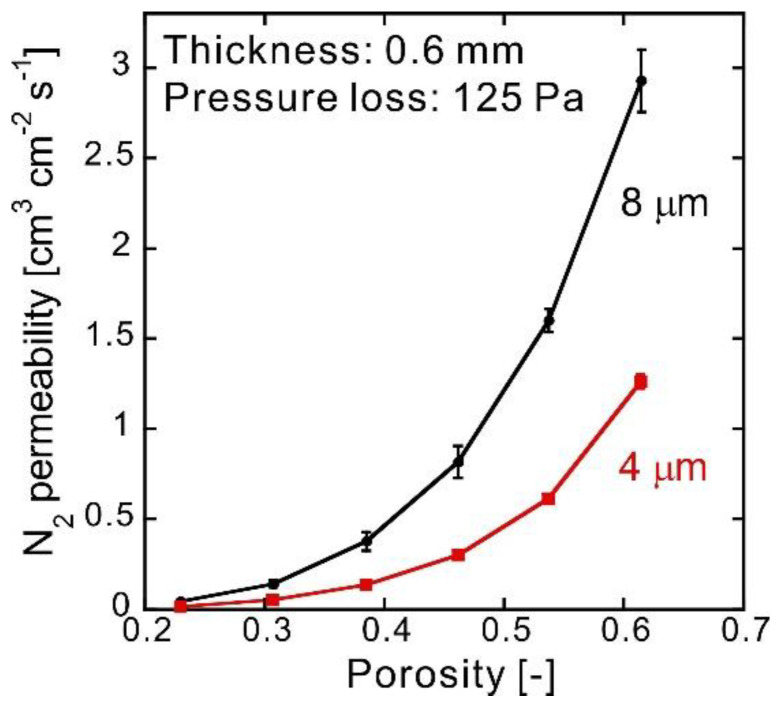
Relationship between porosity and N_2_ permeability when the pressure loss is 125 Pa. Error bars indicate standard deviations.

When the pressure loss was fixed at 125 Pa (the value frequently used in filter characterization), the gas permeability of a 0.600-mm-thick sample could be calculated from the pressure loss at 1 mL min^−1^ Δ*P*, the permeation area *S*, and the thickness *L*. The gas permeability of the sample with the permeation area *S* and thickness *L* was (125 Pa/Δ*P*/60 s min^−1^) [mL s^−1^] at a 125 Pa pressure loss. The inner diameter of gaskets was 8.11 mm, which means that the permeation area *S* was 0.516 cm^2^. Therefore, to convert to the amount of permeation per centimeter squared, it needed to be multiplied by (1 cm^2^/*S*). The sample thickness was approximately 0.600 mm, but since it varied slightly, it was multiplied by (*L*/0.600 mm) to convert it to 0.600 mm. In summary, the gas permeability at a 125 Pa pressure loss and a 0.600 mm thickness was (125 Pa/Δ*P*/60 s min^−1^ × 1.00 cm^2^/*S* × *L*/0.600 mm) [cm^3^ cm^−2^ s^−1^]. The results are plotted in Figure 7, clearly showing that the use of a nonwoven fabric made of thin fibers as the raw material can decrease the gas permeability that, however, increases rapidly along with the porosity. When the fiber diameter was halved, the cross-sectional area of the sample was reduced to a quarter and the number of fibers was quadrupled for the same weight of the sample. The pore diameter, which is the void among the fibers, became smaller. This effect appeared in the difference of N_2_ permeability between fibers of 4 and 8 μm in diameter.

The gas permeability of commercial membrane filters from ADVANTEC (Toyo Roshi Kaisha, Ltd., Chiyoda-ku, Japan) converted to a 0.5 mm thickness with a pressure loss of 125 Pa was 0.3 cm^3^ cm^−2^ s^−1^ for cellulose acetate-type membrane filter (C300A) with a pore size of 3 μm, 0.2 cm^3^ cm^−2^ s^−1^ for a polytetrafluoroethylene-type membrane filter (T300A) with a pore size of 3 μm, and 0.03 cm^3^ cm^−2^ s^−1^ for a polycarbonate-type membrane filter (K800A) with a pore size of 8 μm. The results in Figure 7 show that the CAPC membranes prepared in this study have almost the same potential as a filter material. For further performance improvement, it will be effective to increase the surface area or to use a porous membrane with a gradient pore size. To increase the surface area of the membrane, fabrication using a mold with creases may be an answer. The gradient pore size can be realized by a multi-step press [20].

## 4. Conclusions

The gas permeability of a porous polymer filter prepared via the CAPC method was examined using N_2_. The experiment showed that it increased along with the porosity. By using the same analysis method as for sintered porous materials, we were able to explain gas permeability based on the filter porosity and pore size as follows: The gas permeability decreases due to both pore size and porosity reduction by increasing the degree of compression, which is a feature of the CAPC method. Even for the same porosity, we were able to clarify that the gas permeability of a product prepared using thin fibers decreases because of the formation of small pores. In summary, it became clear that porous material fabricated using the CAPC method featured a decrease in gas permeability that was more than the expected decrease in porosity because the pore diameter became smaller in accordance with the decrease in porosity. Gas permeability is one of the most important properties of filters and these results could offer important design guidelines when using CAPC-fabricated products as filters.

## Figures and Tables

**Table 1:** Experimental results for the samples made of nonwoven fabric with fibers of 8 μm in diameter, obtained via CO_2_-assisted polymer compression.

Number of Sheets [-]	Weight [g]	Thickness [mm]	Porosity [-]	Pressure Loss at 1 mL min^−1^ (Δ*P*) [Pa]	Standard Deviation of Δ*P* [Pa]	Darcian Permeability Coefficient [m^2^]	Pore Diameter [μm]
10	0.219	0.600	0.615	1.38	0.08	2.6 × 10^−12^	17
12	0.263	0.600	0.537	2.53	0.10	1.4 × 10^−12^	13
14	0.306	0.600	0.462	4.99	0.49	7.3 × 10^−13^	10
16	0.350	0.601	0.385	10.9	1.5	3.4 × 10^−13^	7.6
18	0.394	0.601	0.307	19.1	3.8	1.3 × 10^−13^	5.2
20	0.438	0.601	0.230	91.8	8.5	3.9 × 10^−14^	3.4

**Table 2:** Experimental results for the samples made of nonwoven fabric with fibers of 4 μm in diameter, obtained via CO_2_-assisted polymer compression.

Number of Sheets [-]	Weight [g]	Thickness [mm]	Porosity [-]	Pressure Loss at 1 mL min^−1^ (Δ*P*) [Pa]	Standard Deviation of Δ*P* [Pa]	Darcian Permeability Coefficient [m^2^]	Pore Diameter [μm]
10	0.219	0.600	0.614	3.20	0.10	1.1 × 10^−12^	11
12	0.263	0.600	0.537	6.60	0.30	5.4 × 10^−13^	8.2
14	0.306	0.600	0.462	13.5	1.0	2.7 × 10^−13^	6.2
16	0.350	0.601	0.385	30.1	2.4	1.2 × 10^−13^	4.6
18	0.394	0.600	0.306	76.2	4.9	4.7 × 10^−14^	3.2
20	0.438	0.601	0.230	250	37	1.5 × 10^−14^	2.1
